# Conserved Amino Acid Sequence Features in the α Subunits of MoFe, VFe, and FeFe Nitrogenases

**DOI:** 10.1371/journal.pone.0006136

**Published:** 2009-07-03

**Authors:** Alexander N. Glazer, Katerina J. Kechris

**Affiliations:** 1 Department of Molecular and Cell Biology, University of California, Berkeley, California, United States of America; 2 Department of Biostatistics and Informatics, Colorado School of Public Health, University of Colorado Denver, Denver, Colorado, United States of America; Universidad Miguel Hernandez, Spain

## Abstract

**Background:**

This study examines the structural features and phylogeny of the α subunits of 69 full-length NifD (MoFe subunit), VnfD (VFe subunit), and AnfD (FeFe subunit) sequences.

**Methodology/Principal Findings:**

The analyses of this set of sequences included BLAST scores, multiple sequence alignment, examination of patterns of covariant residues, phylogenetic analysis and comparison of the sequences flanking the conserved Cys and His residues that attach the FeMo cofactor to NifD and that are also conserved in the alternative nitrogenases. The results show that NifD nitrogenases fall into two distinct groups. Group I includes NifD sequences from many genera within Bacteria, including all nitrogen-fixing aerobes examined, as well as strict anaerobes and some facultative anaerobes, but no archaeal sequences. In contrast, Group II NifD sequences were limited to a small number of archaeal and bacterial sequences from strict anaerobes. The VnfD and AnfD sequences fall into two separate groups, more closely related to Group II NifD than to Group I NifD. The pattern of perfectly conserved residues, distributed along the full length of the Group I and II NifD, VnfD, and AnfD, confirms unambiguously that these polypeptides are derived from a common ancestral sequence.

**Conclusions/Significance:**

There is no indication of a relationship between the patterns of covariant residues specific to each of the four groups discussed above that would give indications of an evolutionary pathway leading from one type of nitrogenase to another. Rather the totality of the data, along with the phylogenetic analysis, is consistent with a radiation of Group I and II NifDs, VnfD and AnfD from a common ancestral sequence. All the data presented here strongly support the suggestion made by some earlier investigators that the nitrogenase family had already evolved in the last common ancestor of the Archaea and Bacteria.

## Introduction

The ability to perform biological nitrogen fixation is restricted to Bacteria and Archaea. This trait appeared early in the evolution of prokaryotes, over a billion years ago, perhaps as long as 2.5 bya [1]. All N_2_-fixing organisms studied depend on a nitrogenase for the conversion of atmospheric nitrogen to ammonia and all have the genes encoding the subunits of molybdenum-containing nitrogenase. This enzyme is an α_2_β_2_ tetramer, where the two α subunits (the FeMo-protein) catalyze the ATP-dependent reduction of N_2_ to NH_3_, and β_2_ (referred to as the Fe-protein) mediates the coupling of ATP hydrolysis to electron transfer and is the only known electron donor that can support reduction by the MoFe protein [2]. The α subunits each contain a MoFe_7_S_9_ metal cluster (the FeMo-cofactor).

Recently, we examined the NifD, NifK, NifE, and NifN sequences from a limited set of complete genomes of diazotrophs [3]. *NifD*, and *nifK*, respectively, encode the α- and β-subunits of the MoFe-nitrogenase, whereas *nifE* and *nifN* are required for the synthesis of the MoFe cofactor. We found that the four sets of sequences fell consistently into two groups based on BLAST scores, distinctive patterns of conserved covariant amino acid residues [4] and, in the case of NifD, different patterns of invariant residues in the sequences flanking the Cys-α275 and His-α442 (*Azobacter vinelandii* sequence numbering) residues that attach the FeMo cofactor to NifD [5].

As noted above, the *nifD* gene encodes the FeMo-nitrogenase α subunit. In addition to *nifD*, some organisms contain genes that encode alternative nitrogenases, based on vanadium and iron, or on iron alone that are expressed when Mo is unavailable [6]. *VnfD* encodes VnfD (VFe-protein alpha-subunit), and *AnfD* encodes AnfD (FeFe-protein alpha-subunit). VnfD and AnfD show strong homology to NifD. In these proteins, V and Fe, respectively, occupy the place of Mo in the FeMo cofactor. The available data indicate that other than these metal substitutions, the structures of the three cofactors and their protein environment are very similar [7]. Since X-ray structures are not available for VnfD or AnfD containing nitrogenases, this is an assumption based on amino acid sequence homology.

In this report, we present the data and the conclusions resulting from an extension of our earlier study to a large set of NifD sequences, as well as to smaller sets of sequences of alternative nitrogenases. Our earlier study was aimed at the unambiguous assessment of the potential contribution of lateral gene transfer to the spread of nitrogenase genes among diverse prokaryotes. The present study provides a more rigorous test of the validity of the distinctions between the structural features of Group I and II NifD sequences that we had reported earlier, provides additional details of the evolutionary history of nitrogenases, and attempts to generate hypotheses about structure-function relationships in nitrogenase and the evolution of this family of metalloenzymes.

## Results

### Two Groups of NifD Sequences

We reported earlier, based on the analysis of sequences from fourteen organisms of the proteins encoded by *nifD, K, E, and N*, that these sequences fall into two groups distinguished by distinctive characteristics of their amino acid sequences [3]. In this study, focusing solely on NifD, we included complete sequences available as of the end of 2008 on GenBank. In many cases, multiple NifD sequences are available for different strains from the same genus and species, as well as multiple NifD sequences for different species within a genus. We compared the sequences for a number of such closely related organisms and found little variation in these instances. In general, each NifD sequence included among 54 sequences examined in the current study is a typical representative of the known NifD sequences from organisms in that genus. In a few specific cases, we present data separately on additional closely related NifD sequences.

**Figure 1:**
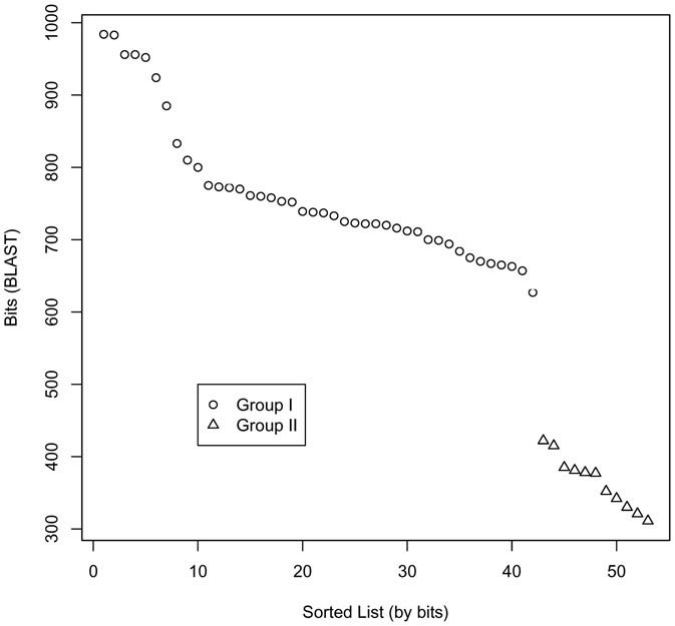
Plot of BLAST bit scores for NifD sequences relative to Nostoc sp. NifD. The Nostoc sp. NifD sequence was used as a query sequence for BLAST against all Group I (triangle) and II (circle) sequences from Table 1 and 2. Each point represents a BLAST result (in bits on the y-axis) and all points are sorted on the x-axis by the bit value.

**Figure 2:**
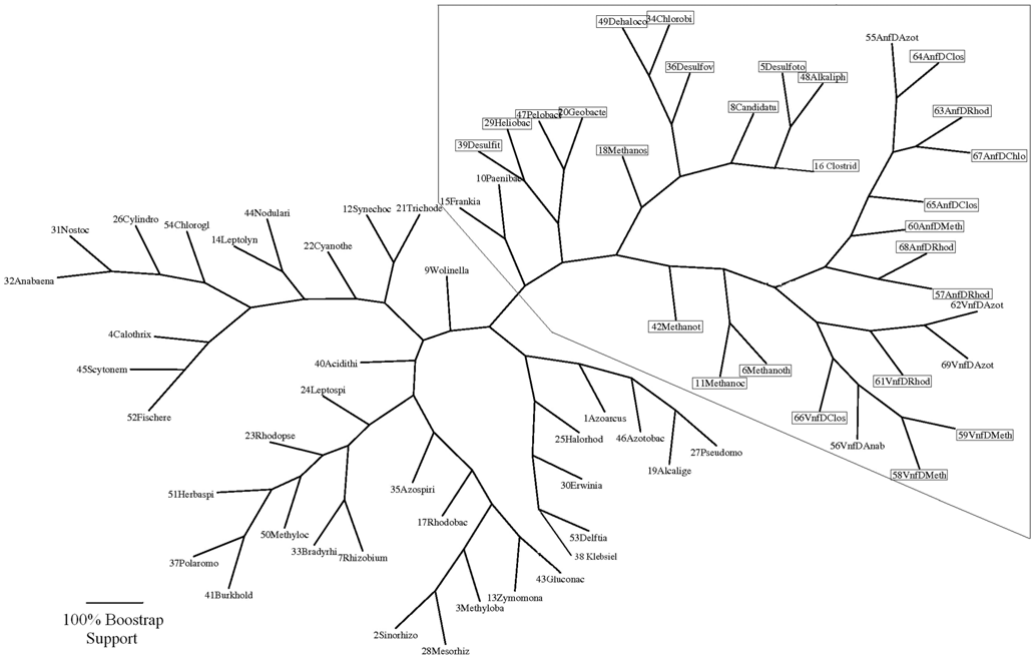
Phylogeny of nitrogenases. The phylogeny is constructed based on the sequences and alignment in Figure S1. See the legend in Table S4 for leaf labels, which have been truncated to 10 letters. The outlined region is summarized in Figure 3. The branch lengths indicate the percentage of bootstrap samples that support the branching pattern. Strict anaerobes are outlined in boxes.

The analysis of the 54 NifD sequences, listed in Tables 1 and 2, confirmed and extended our earlier finding that NifD sequences fall into two structurally distinct groups, as indicated by the BLAST scores shown graphically in Figure 1. Table 3 provides the lineages of the organisms with Group I and II NifD sequences. Tables S1 and S2 list the lineages for each of the strains in Tables 1 and 2. Group I sequences were present in all five classes of proteobacteria (distributed among 19 families), in several classes of cyanobacteria, including both unicellular and filamentous organisms, and in Actinobacteria, Bacilli, Clostridia, and Nitrospira. There are no archaeal sequences in Group I. Organisms with a Group I NifD sequence included all nitrogen-fixing aerobes examined, as well as some organisms able to tolerate low levels of oxygen (e.g., *Clostridium kluyveri*), and strict anaerobes (e.g., *Heliobacterium chlorum*, *Desulfobacterium hafniense*). Occurrence of Group II sequences was limited to organisms in a small group of genera of methanogenic Archaea, and six genera of eubacteria. All of these organisms were strict anaerobes. As shown in Figure 2, Group II sequences, whether archaeal or bacterial, clustered deep in the tree of the NifD sequences. Undoubtedly, the number of additional classes of NifD-containing microorganisms with Group I or II NifD sequences will continue to grow in years to come.

**Table 1:** Group I NifD sequences surrounding the His and Cys residues that attach the MoFe cofactor to the protein.

Organism	GI*	His-442**	Cys-275**
Invariant residues in Class 1 sequences		FRQMHSWDY	--------HCYRS-----Y
*Azotobacter vinelandii*	158358	**FRQMHSWDY**	LNLV**HCYRS**MN**Y**
*Frankia* sp.	21930294	**FRQMHSWDY**	LNLI**HCYRS**MN**Y**
*Azoarcus* sp.	119669243	**FRQMHSWDY**	LNLL**HCYRS**MN**Y**
*Alcaligenes faecalis*	1183862	**FRQMHSWDY**	LNLV**HCYRS**MN**Y**
*Pseudomonas stutzeri*	146281711	**FREMHSWDY**	LNLV**HCYRS**MN**Y**
*Klebsiella pneumoniae*	43847	**FRQMHSWDY**	LNLV**HCYRS**MN**Y**
*Delftia tsuruhatensis*	45269095	**FRQMHSWDY**	LXLV**HCYRS**MN**Y**
*Erwinia carotovora*	50121879	**FRQMHSWDY**	LNLV**HCYRS**MN**Y**
*Halorhodospira halophila*	62122622	**FRQMHSWDY**	LNLL**HCYRS**MN**Y**
*Rhizobium* sp.	224328	**FRQMHSWDY**	LNIL**HCYRS**MN**Y**
*Bradyrhizobium japonicum*	126220453	**FRQMHSWDY**	LNIL**HCYRS**MN**Y**
*Methylococcus capsulatus*	53802573	**FRQMHSWDY**	LNVL**HCYRS**MN**Y**
*Polaromonas napthalenivorans*	121605243	**FRQMHSWDY**	LNVL**HCYRS**MN**Y**
*Burkholderia xenovorans*	91778640	**FRQMHSWDY**	LNVL**HCYRS**MN**Y**
*Leptospirillum ferrooxidans*	31747711	**FRQMHSWDY**	LNVL**HCYRS**MN**Y**
*Herbaspirillum seropedicae*	6093493	**FRQMHSWDY**	LNVL**HCYRS**MN**Y**
*Rhodopseudomonas palustris*	39937677	**FRQMHSWDY**	LNIL**HCYRS**MN**Y**
*Sinorhizobium medicae*	150378166	**FRQMHSWDY**	LNLI**HCYRS**MN**Y**
*Mesorhizobium loti*	20804122	**FRQMHSWDY**	LNLI**HCYRS**MN**Y**
*Methylobacterium* sp.	149123939	**FRQMHSWDY**	LNLI**HCYRS**MN**Y**
*Gluconacetobacter diazotrophicus*	4103974	**FRQMHSWDY**	LNLI**HCYRS**MN**Y**
*Zymomonas mobilis*	56552720	**FRQMHSWDY**	MNLI**HCYRS**LN**Y**
*Rhodobacter sphaeroides*	77464109	**FRQMHSWDY**	LNLI**HCYRS**MS**Y**
*Azospirillum brasilense* ***	142417	**FRQMHSWDY**	VNLI**HSYRS**MN**Y**
*Scytonema* sp.	30983593	**FRQMHSWDY**	LVLI**HCYRS**MN**Y**
*Fischerella muscicola*	24637390	**FRQMHSWDY**	LVLI**HCYRS**MN**Y**
*Calothrix desertica*	24637368	**FRQMHSWDY**	LVLI**HCYRS**MN**Y**
*Chlorogloeopsis fritschii*	24637386	**FRQMHSWDY**	LVLI**HCYRS**MN**Y**
*Cylindrospermum majus*	30983589	**FRQMHSWDY**	LVLI**HCYRS**MN**Y**
*Nostoc* sp.	24637372	**FRQMHSWDY**	LVLI**HCYRS**MN**Y**
*Anabaena* sp.	223741	**FRQMHSWDY**	LVLI**HCYRS**MN**Y**
*Nodularia spumigena*	24637384	**FRQMHSWDY**	MNLI**HCYRS**MN**Y**
*Leptolyngbya boryana*	228688	**FRQMHSWDY**	LNLV**HCYRS**MN**Y**
*Synechococcus* sp.	866071919	**FRQMHSWDY**	LNLI**HCYRS**MN**Y**
*Trichodesmium erythraeum*	3372146	**FRQMHSWDY**	LNLI**HCYRS**MN**Y**
*Cyanothece* sp.	2197063	**FRQMHSWDY**	LNLI**HCYRS**MN**Y**
*Wolinella succinogenenes*	34483460	**FRQMHSWDY**	LNLL**HCYRS**MN**Y**
*Acidithiobacillus ferrooxidans*	154639	**FRQMHSWDY**	LNLL**HCYRS**VN**Y**
*Paenibacillus massiliensis*	62512189	**FRQMHSWDY**	LNLI**HCHRS**MN**Y**
*Heliobacterium chlorum*	62751062	**FRQMHSWDY**	LNLI**HCYRS**MN**Y**
*Desulfitobacterium hafniense*	1096448676	**FRQMHSWDY**	LNLI**HCYRS**MN**Y**
*Geobacter sulfurreducens*	39997913	**FRQMHSWDY**	LNLI**HCYRS**MN**Y**

* GI is the NCBI Geninfo identifier.

** The residue numbers shown, His-442 and Cys-275 refer to the sequence of *Azotobacter vinelandii* (GI: 758358) NifD. In the nitrogenase multiple sequence alignment (Figure S1) the corresponding residue numbers are His-562 and Cys-330. Invariant residues are shown in bold face.

*** *Azospirillum brasilense* NifD (GI:142417) sequence shows a Ser residue in the position of the invariant Cys residue. Examination of the entire sequence suggests that this may either be a sequence error or that the sequence is that of a mutant NifD.

**Table 2:** Group II NifD sequences surrounding the His and Cys residues that attach the MoFe cofactor to the protein.

Organism	GI*	His-442**	Cys-275**
Invariant residues in Class II sequences		--------HSY--	L-------C--RS----Y
Archaea			
*Methanothermobacter thermautotrophicus*	1854556	CILI**HSY**EN	**L**SLVR**C**Q**RS**AN**Y**
*Methanococcus maripaludis*	46397844	SVMI**HSY**EN	**L**SLVR**C**Q**RS**AT**Y**
*Methanothermococcus thermolithotrophicus*	128245	TINS**HSY**EE	**L**NIVH**C**Q**RS**AE**Y**
*Methanosarcina barkeri* (+)	508282	SRQI**HSY**DY	**L**SILV**C**H**RS**IN**Y**
*Candidatus Methanoregula boonei* (+)	154150688	AKQM**HSY**DY	**L**NLVQ**C**H**RS**IN**Y**
Bacteria			
*Desulfotomaculum reducens* (+)	134300651	AKQL**HSY**DY	**L**NLVQ**C**H**RS**IN**Y**
*Alkaliphilus metalliredigens* (+)	150391258	SKQL**HSY**DY	**L**NLVQ**C**H**RS**IN**Y**
*Clostridium beijerinckii* (+)	150016874	SRQL**HSY**DY	**L**NLVQ**C**H**RS**IN**Y**
*Chlorobium tepidum* (+)	21674356	LKQL**HSY**DY	**L**NVIM**C**H**RS**IN**Y**
*Dehalococcoides ethenogenes* (+)	57234132	CLQL**HSY**DY	**L**NLVM**C**H**RS**IN**Y**
*Desulfovibrio vulgaris* (+)	46562234	CKQL**HNY**DY	**L**NLVM**C**H**RS**IN**Y**

* GI is the NCBI Geninfo identifier.

** The residue numbers shown, His-442 and Cys-275, refer to the sequence of Azotobacter vinelandii NifD (GI: 758358). In the nitrogenase multiple sequence alignment (Figure S1) the corresponding residue numbers are His-562 and Cys-330. Invariant residues are shown in bold face.

(+) Indicates the presence of the 51-residue insertion described in the text.

**Table 3:** Lineages of Archaea and Bacteria with the NifD sequences listed in Tables 1 and 2.

Class	Family
**Group I**	
Actinobacteria	Frankineae
Bacilli	Paenibacillaceae
Clostridium	Peptococcaceae
Nitrospira	Nitrospiraceae
α-Proteobacteria	Acetobacteraceae, Bradyrhizobiaceae, Methylobacteraceae,
	Phyllobacteriaceae, Rhizobiaceae, Rhodospirillaceae
	Rhodobacteriaceae
β-Proteobacteria	Alcaliginaceae, Burkholderiaceae, Comamonadaceae,
	Oxalobacteraceae, Rhodocyclaceae
γ-Proteobacteria	Acidithiobacillaceae, Ectothiorhodospiraceae
	Enterobacteriaceae, Pseudomonadaceae
δ-Proteobacteria	Geobacteriaceae, Pelobacteriaceae
ε-Proteobacteria	Campylobacteraceae
Chroococcales	*NA*
Nostocales	Nostocaceae, Rivulariaceae, Scytonemataceae
Oscillatoriales	*NA*
Stigonematales	*NA*
**Group II**	
**Archaea**	
Methanobacteria	Methanobacteriaceae
Methanococci	Methanococcaceae
Methanomicrobia	Uncertain
Methanosarcina	Methanosarcinaceae
**Eubacteria**	
Clostridia	Clostridiaceae, Peptococcaceae
Methanomicrobia	Uncertain
Chlorobia	Clorobiaceae
Dehalococcoidetes	Dehalococcoides
δ-Proteobacteria	Desulfovibrionaceae

* Lineage data were obtained from the NCBI Taxonomy Browser at http://www.ncbi.nlm.nih.gov/Taxonomy/Browser/wwwtax.cgi. Information on the lineage for the each of the organisms examined in this study is provided in the Supplementary Material in Tables S1 and S2.

### Invariant Residues in the Sequences Flanking the Cys and His Ligands to FeMo cofactor

A multiple sequence alignment of the 54 NifD sequences and 15 alternative nitrogenases discussed below is shown in the Supplementary Material in Figure S1. This alignment facilitated the comparison of the sequences flanking the conserved Cys and His residues that attach the FeMo cofactor to NifD. In the multiple sequence alignment, these residues are Cys-333 and His-565 (Figure S1). However, throughout this paper, we use the numbering for *Azotobacter vinelandii* NifD (GI: 758358), because this is the only nitrogenase for which a high resolution 1.16 Å X-ray structure is available [8]. In this sequence the corresponding residues are Cysα-275 and Hisα-442. Cys-275 coordinates Fe1 and His-442 along with homocitrate coordinate Mo in the FeMo cofactor. Fe1 is the iron atom in the FeMo cofactor located at the opposite apex of the cofactor to that occupied by the Mo atom [8] *A. vinelandii* NifD is a member of Group I. In the Group II NifD of *Clostridium pasteurianum* FeMo-nitrogenase, whose X-ray crystal structure was determined at a 3.0 Å resolution, the corresponding residues are Cysα-262 and Hisα-482 [9].

In accord with the finding in our earlier study of a much smaller set of NifD sequences, the patterns of conserved residues in the twelve-residue sequence that includes Cys-275 or in the nine-residue sequence centered on His-442 allow unambiguous assignment of a NifD sequence to either Group I or Group II (Tables 1 and 2). The nine-residue sequence is entirely conserved in all Group I sequences examined. The resulting Group assignments are in full accord with those based on BLAST scores (Figure 1).

### Invariant Residues in the Sequences Flanking the Cys and His Ligands to VFe cofactor in VnfD and FeFe cofactor in AnfD

In the nitrogenase vanadium-iron protein α subunit (VnfD), and in nitrogenase iron-iron protein α subunit (AnfD), V and Fe, respectively, are believed to occupy the place of Mo in the cofactor. Whereas the three types of nitrogenase α chains are homologous over the full length of these polypeptides, VnfD and AnfD are much more similar to each other than they are to NifD in the same organism. For example, in *Clostridium kluyveri*, VnfD and AnfD share 61% identity, whereas VnfD and NifD share 34% identity, and AnfD and NifD share 32%. In *Azotobacter vinelandii*, VnfD and AnfD share 55% identity, whereas VnfD and NifD share 33% identity, and AnfD and NifD only 32%. In 1996, Eady [6] noted that NifD, VnfD, and AnfD could be distinguished by inspection of the amino acid sequences flanking the His residue that acted as a ligand to the metal cofactor (See Table 1 in ref. 6), and proposed that “the determination of the sequence in this region would appear to allow the assignment of the types of Mo-independent nitrogenase in an organism from DNA sequences.”

Table 4 presents data on VnfD and AnfD sequences that include His-442 and Cys-275, and compares the patterns of invariant residues in these sequences with those in the corresponding sequences in Group I and Group II NifD (see Tables 1 and 2). For NifD sequences, assignment of a particular sequence either to Group I or to Group II on the basis of these patterns is unambiguous. Because only a relatively small number of sequences of alternative nitrogenases have been reported to date, the distinction between VnfD and AnfD based on the same basis is less secure. In the 12-residue sequence that includes VnfD Cys-275, there are 9 invariant residues. All 12 residues are invariant in the corresponding AnfD sequences. VnfD and AnfD differ at position 274 and 280 within this 12-residue sequence containing Cys-275. If one were to rely entirely on the 9-residue sequence that includes His-442 to discriminate between VnfD and AnfD, the decision would rest solely on the presence of Gly-441 in the VnfD sequence and the Leu-439 in the AnfD sequence. However, the availability of much larger numbers of VnfD and AnfD is needed to allow a greater level of confidence in the invariable occurrence of signature residues.

**Table 4:** Conserved residue patterns in nitrogenase VFe α subunit (VnfD), and nitrogenase FeFe α subunit (AnfD), and comparison with the corresponding patterns in Group I and II NifDs.

Protein	GI*	Organism	Residues surrounding †	
			His α-442	Cys α-275
**Invariant residues in VnfD**			**Y-NGH-YHN**	**L**-**V**-**NCARS**-**GY**
VnfD	67154938	*Azotobacter vinelandii*	**Y**V**NGH**G**YHN**	**L**N**V**V**NCARS**S**GY**
VnfD	138885	*Azotobacter chroococcum*	**Y**V**NGH**G**YHN**	**L**N**V**V**NCARS**S**GY**
VnfD	19915055	*Methanosarcina acetivorans*	**Y**I**NGH**A**YHN**	**L**N**V**V**NCARS**A**GY**
VnfD	8099626	*Methanosarcina barkeri*	**Y**I**NGH**A**YHN**	**L**N**V**V**NCARS**A**GY**
VnfD	153954372	*Clostridium kluyveri*	**Y**I**NGH**A**YHN**	**L**S**V**V**NCARS**A**GY**
VnfD	39648301	*Rhodopseudomonas palustris*	**Y**V**NGH**A**YHN**	**L**S**V**V**NCARS**A**GY**
VnfD	416166	*Anabaena variabilis*	**Y**V**NGH**G**YHN**	**L**N**V**T**NCARS**A**GY**
**Invariant residues in AnfD**			**YLN-H-YHN**	**LNVLECARSAEY**
AnfD	113854	*Azotobacter vinelandii*	**YLN**A**H**A**YHN**	**LNVLECARSAEY**
AnfD	20090074	*Methanosarcina acetivorans*	**YLN**I**H**G**YHN**	**LNVLECARSAEY**
AnfD	84028173	*Clostridium hungatei*	**YLN**A**H**A**YHN**	**LNVLECARSAEY**
AnfD	146345888	*Clostridium kluyveri*	**YLN**A**H**A**YHN**	**LNVLECARSAEY**
AnfD	83592730	*Rhodospirillum rubrum*	**YLN**A**H**A**YHN**	**LNVLECARSAEY**
AnfD	728856	*Rhodobacter capsulatus*	**YLN**A**H**A**YHN**	**LNVLECARSAEY**
AnfD	221369728	*Rhodobacter sphaeroides*	**YLN**A**H**A**YHN**	**LNVLECARSAEY**
AnfD	193215536	*Chloroherpeton thalassium*	**YLN**A**H**A**YHN**	**LNVLECARSAEY**
Invariant residues in Group I NifD signature sequences			**FRQMHSWDY**	----**HCYRSMNY**
Invariant residues in Group II NifD signature sequences			----**HSY**-**-**	**L**----**C**-**RS--Y**
Invariant residues in VnfD signature sequences			**Y-NGH-YHN**	**L**-**V**-**NCARS**-**GY**
Invariant residues in AnfD signature sequences			**YLN-H-YHN**	**LNVLECARSAEY**

* NCBI GenInfo Identifier

† Conserved residues neighboring His **α**-442 and Cys **α**-275 (using *Azotobacter vinelandii* sequence numbering) within Group I and Group II NifD, VnfD, and AnfD proteins are shown in bold face.

### Distribution of a 51-Residue Insertion among NifD Sequences

The presence of a ∼50-residue insertion in the carboxyl-terminal portion of certain NifD sequences was noted in several earlier studies [10]–[12]. The current study showed that this insertion occurred only in Group II NifDs. These organisms include methanogenic Archaea as well as representatives from a number of classes of Bacteria (Tables 2 and S2). Using the numbering of the sequence of Group I *Azotobacter vinelandii* NifD as a reference, the insertion is located between residues 390 and 391, which spans positions 454–510 in the multiple sequence alignment (Figure S1). Among the Group II NifD sequences listed in Table 2, all six bacterial sequences contain the insertion. However, it is present in only two of the archaeal methanogen sequences, *Candidatus Methanoregula boonei* and *Methanosarcina barkeri*. In addition, we established that *Methanosarcina acetivorans* and *Methanosarcina mazei* also contained Group II NifDs with the insertion.

We were struck by the observation that the insertion was present in *Clostridium beijerinckii* NifD, but absent in *Desulfitobacterium hafniense* NifD (found in Group I). Both of these organisms are within the class Clostridia and the family Peptococcaceae. For a broader assessment of *Clostridium* strains, we examined NifD in *C. acetobutylicum* ATCC 824, *C. beijerinckii* NRRL B593 and NCIB8052, *C. butyricum* 5521, *C. kluyveri* DSM 555, and *C. pasteurianum* W5, and found all the sequences to belong to Group II NifD and to contain the insertion. The GenBank ID numbers of these proteins, and the sequences flanking the His and Cys residues, are provided in Table S3.

The insertion is absent from all VnfD and AnfD sequences examined in this study. Several organisms with Group II NifD sequences that contain the insertion also have the genes for the alternative nitrogenases (Table 4). For example, *Clostridium kluyveri* NifD contains the insertion whereas there is no insertion in either VnfD or AnfD in this strain [13]. Moreover, examination of pairwise alignments of *C. kluyveri* NifD, VnfD, and AnfD, clearly show continuous full-length homology between these three polypeptide, where the insertion interposes between two segments of VnfD, or AnfD that together comprise the full sequence of each of these alternative nitrogenases (data not shown). The location of the insertion as seen in these comparisons is the same as that seen in the alignment of a Group I nitrogenase, such as *A. vinelandii* NifD with *C. kluyveri* NifD.

### Covariant Residues in Group I and II NifDs, VnfD, and AnfD

The patterns of covariant residues [4] in the 69 sequences examined here are shown in Table 5 and allow some general conclusions. Thirty four perfectly conserved residues, distributed along the full length of the four nitrogenase α subunits, confirm unambiguously that these polypeptides are derived from a common ancestral sequence. However, there is no indication of a sequential variation in the patterns of covariant residues that would give indications of an evolutionary pathway leading from one type of nitrogenase to another. Rather the data are consistent with a radiation of Group I and II NifDs, VnfD and AnfD from a common ancestral sequence.

**Table 5:** Patterns of covariant residues in the nitrogenases.

Sequences	Residues
**NifD/AnfD/VnfD (n = 69)**	G86 C93 V101 D108 H114 P116 G118 C119 G165 L170 G212 D213 G238 G241 Q244 S245 G247 H248 N288 G305 G314 R336 S337 Y340 G360 I388 G419 F444 H446 D526 E532 G548 K557 H566
**NifD I (n = 43)**	E203 L244 D251 L304 E353 Y389 A427 T451 F560 W566
**NifD II (n = 11)**	*NA*
**AnfD/VnfD (n = 15)**	F189 R197 M198 I251 Q288 A333 E342 R351 D353 F359 W399 W414 L420 K441 G550 K554 Y560 H567 D600

In addition to the 34 residues, AnfD and VnfD share a further 19 covariant residues unique to these polypeptides, while Group I NifDs share 10 additional covariant residues in common. In sharp contrast, Group II NifDs have no additional covariant residues. These results emphasize the close structural relationship of VnfD and AnfD sequences and the high level of variability among Group II NifDs.

## Discussion

The wealth of information in protein sequence databases is large and growing rapidly. Such information is particularly valuable, and potentially rich in opportunities for new insights, where for each sequence, information is available on the ecology, physiology, and biochemistry of the source organism, and even more so where the complete genome has been sequenced. These criteria are met by many of the sequences examined in this study. The focus here has been on features of primary structure that are strictly conserved in subgroups of sequences generated by examination of BLAST scores and other broad measures of relatedness. To have any chance of formulating plausible explanations, or hypotheses, concerning the role(s) of such conserved features, detailed information on the structure and function of the subject macromolecule, its interactions with other macromolecules and/or small molecules, is invaluable. The choice of the subjects of this study, the α subunits of nitrogenase and of alternative nitrogenases, is motivated by their central role in a reaction of great importance to biology, wealth of information accumulated over decades of study of diazotrophs, of the process of nitrogen fixation and its regulation, and of nitrogenase proteins.

Nitrogen-fixing organisms are found among denizens of the microbial world that occupy a great variety of ecological niches encompassing environments that differ in macro- and micronutrient composition, in trace metal availability, in pH, salinity, oxygen concentration, temperature (the current upper bound for N_2_ fixation is 92°C), and so on. Moreover, because of the patchiness of the environment on the large, and, frequently even on a small scale with respect to such parameters, microorganisms confront the needs of adjusting to microhabitats that may be only inches apart, but that make significant distinctive demands on their adaptability. The groups of polypeptides examined in this study, are derived from a common ancestral gene, are the present day exemplars of an evolutionary history spanning billions of years, and come from organisms with a range different histories of exposure to distinctive selective pressures of all kinds. With recognition of these factors, it is evident that strictly invariant features of the sequence of a subgroup of nitrogenases play an important role in the structure and/or function of the protein molecule itself.

This study has focused on the following aspects of the sequences of nitrogenase and of the alternative nitrogenases: (i) the distribution among diazotrophs of a ∼50-residue insertion within the carboxyl terminal half of the NifD, (ii) the patterns of conservation of residues in sequences that surround the His residue that serves to coordinate the Mo in the FeMo-cofactor in NifD and the His residues in the corresponding position in VnfD and AnfD, and (iii) the patterns of conservation of residues in sequences that surround the Cys residue that serves to coordinate the Fe1 in the FeMo-cofactor in NifD and the Cys residues in the corresponding position in VnfD and AnfD.

Examination of the multiple sequence alignment of the NifD sequences (Figure S1) shows that the insertion sequence is present in exactly the same place in a subset of the Group II NifD sequences listed in Table 2. To support the belief that the NifD sequence of the insertion-containing strains listed in Table 2 is generally representative of the NifD sequences of other members of the genus they represent, we examined the NifD sequences of two other *Methanosarcina* species, *M. mazei* and *M. acetivorans* and confirmed that these sequences also contain the insertion. Because many complete genome sequences of clostridia are available, we chose *Clostridium beijerinckii* NifD as representative of insertion-containing NifD sequences, and examined the sequences of five additional *Clostridium* species (Genbank IDs listed in Table S3). All of these sequences contained the insertion in the identical location.

Whereas organisms with insertion-containing NifD sequences are found both among methanogenic Archaea and in several families of Bacteria (Tables 2 and 3), there are no archaeal NifD sequences among Group I NifD sequences (Tables 1 and 3). It is striking that *the insertion is absent* from all the bacterial Group I NifD sequences, and is also absent from both the archaeal and bacterial VnfD or AnfD sequences listed in Table 4.

With no exception, in organisms with a NifD that has the insertion, and whose genome also encodes one or both VnfD and AnfD, the insertion was absent from all of the alternative nitrogenase sequences examined. We found no literature reports of an insertion-containing VnfD or AnfD sequence. We return to this point below.

In *C. pasteurianum* nitrogenase, the insertion includes residues α375-α430 [9]. The FeMo-cofactor is located beneath a wide, shallow cleft between the three domains of NifD. A portion of the insertion, residues α383-α397 (*C. pasteurianum* sequence numbering), lies above the cleft [9]. In that location it might influence access of small molecules to the cofactor, but there is no information bearing on this possibility. The strict conservation of the location and length of this insertion in the archaeal and bacterial families listed in Table S2, in the face of selection pressures over much of the history of the evolution of nitrogenases, argues that a role remains to be found for this additional segment of polypeptide.

We next consider the conserved patterns of residues that surround the conserved His-442 and Cys-275 residues (using *A. vinelandii* sequence numbering) that attach the FeMo-cofactor to NifD. The invariant nine-residue sequence that includes His-442 unambiguously distinguishes Group I NifD sequences from Group II NifD, VnfD, and AnfD sequences. A striking diagnostic marker is the tryptophan residue, W-442. In Group II NifD, VnfD, and AnfD, there is an invariant tyrosine residue, Y-442, in the corresponding position (Table 4). The invariant residue pattern in Group I NifD sequence surrounding Cys-275 also distinguishes Group I NifD from Group II NifD (Tables 1 and 4) and also from VnfD and AnfD. It is notable, however, that the Group I and II NifD, VnfD, and AnfD sequences all show the invariant residue pattern C-275, R-277, S-278, Y-281. With respect to the very limited information represented by these patterns of invariant residues, Group II NifD, VnfD, and AnfD appear closely related. Group I NifD sequences are distinctly different. The future sequencing of many additional VnfD and AnfDs may well change this clustering, The one finding that is highly unlikely to be negated by new data is the uniqueness of the invariant nine-residue sequence encompassing His-442, a hallmark of Group I NifD sequences. Since Group I NifD sequences have a far wider distribution among Bacteria than the Group II NifD sequences, the basis of an implied selective advantage attributable to this nine-residue invariant sequence merits careful consideration.

The distribution of sequences in the consensus tree, the distinctive features in the sequence data that emerged from this study, and additional considerations, provide strong support for a suggestion advanced by some earlier investigators that the nitrogenase family had already evolved in the last common ancestor of the Archaea and Bacteria [11], [14], [15]. with this scenario.

**Figure 3:**
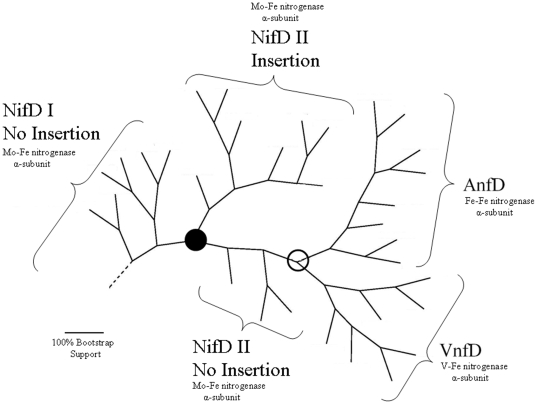
Summary of nitrogenase phylogeny. Adaptation of Figure 2 summarizing major nitrogenase sub-classes. Nitrogenase types are indicated under each sub-class. The open circle indicates the location of the putative ancestral gene. The filled circle indicates the point in the tree beyond which all the sequences are Group I NifDs.

(1) *Distribution of sequences from anaerobes on the consensus tree.* Sequences from strict anaerobes are indicated on the consensus tree in Figure 2 by boxed labels. These sequences are clustered in the portion of the tree delimited by a box outline, and illustrated further in Figure 3. This portion of the tree encompasses all of the Group II NifD, VnfD, and AnfD sequences. Among the alternative nitrogenase sequences, there are a few sequences from aerobes (*Azotobacter*, *Anabaena*), but their presence may reflect outcomes of lateral gene transfer [3], [11]. All of the archaeal sequences also lie within this portion of the consensus tree.

All of the sequences on the tree, beyond the point indicated by a filled circle are Group I NifD. It is noteworthy, that the branch immediately beyond the filled circle includes the sequences of the four strict anaerobes among the organisms that have Group I NifD sequences (Figures 2 and 3). Two of these (*Desulfitobacterium* and *Heliobacterium*) are Clostridia and two (*Pelobacter* and *Geobacteri*) are Proteobacteria.

It is generally accepted that the advent of biological nitrogen fixation was an early event in the evolution of life on Earth, and certainly occurred at a time when the atmosphere contained very little, if any, oxygen. Nitrogenases are rapidly inactivated by oxygen. Aerobic nitrogen fixers utilize a variety of protective mechanisms that serve to slow down the inactivation of nitrogenase. Understandably, strictly anaerobic nitrogen fixers have not evolved such mechanisms. The overwhelming predominance of strict anaerobes on the portion of the tree illustrated in Figure 3 supports the view that all four nitrogenase types evolved in an anaerobic atmosphere in the ancestor(s) of these organisms.

(2) *Implications of the distribution of the ∼50-residue insertion among nitrogenase α chain sequences.* The exclusive occurrence of the insertion in a subset of NifD sequences, but its complete absence from VnfD and AnfD, establishes that the ancestral gene duplication events that led to the evolution of the different types of nitrogenase α chains took place before the 50-residue insertion into an ancestral NifD gene, most likely in a single organism.

(3) *Patterns* of *invariant residues in the sequences that surround the conserved His-442 and Cys-275 residues in VnfD and AnfD.* All VnfD and AnfD sequences examined share the patterns of invariant residues in the sequences that surround their conserved His-442 and Cys-275 residues. In the alternative nitrogenases, these patterns remain the same irrespective of whether the VnfD and/or AnfD is expressed in an organism whose genome expresses a Group II NifD or a Group I NifD (Table 4). This observation is consistent with a radiation of Groups I and II NifDs, VnfD, and AnfD from a common ancestral sequence located at the branch point marked in Figure 3 by an open circle.

Finally, nitrogenase-catalyzed reduction of nitrogen to ammonia is accompanied by the production of hydrogen. Under optimal conditions, the product ratio of hydrogen to ammonia is 1 for MoFe-nitrogenase (the most efficient nitrogenase), 4 for VFe-nitrogenase, and very much higher for FeFe-nitrogenase [6], [7], [16]. Expression of either VnfD when Mo is limiting, or of AnfD when both Mo and V are limiting, indicates that retention of the genes for the less efficient nitrogenases still confers an evolutionary advantage on numerous organisms that occur in niches where Mo concentrations are very low (e.g., [17], [18]). This points to the possibility that the early evolution of nitrogen fixation may have taken place in terrestrial settings where the availability of trace metals is known to be patchy.

## Methods

### Sequence Collections

Using the NCBI search engine (10/2007 and updated 01/2009), the protein NifD was searched with the query word “nifd” which returned 1075 entries. All 1075 “nifd” entries were downloaded in the fasta format and 121 full-length sequences (length of more than 400 residues), with organismal information (i.e., not labeled “uncultured” and “unidentified”) were retained. Manual checks also verified that these were NifD proteins. Because many sequences were from related organisms, a smaller set of 54 NifD sequences based on representatives from each genus were examined. VnfD and AnfD sequences were searched with the query words “vnfd” and “anfd” respectively at NCBI. Because of the small number of these alternative nitrogenases, sequences from multiple species within a genus were retained for analysis for a final set of 15 alternative nitrogenases. The complete set of nitrogenases analyzed in this study contains 69 (54+15) sequences. Information for these sequences is listed in Tables 1, 2 and 4 and a multiple protein sequence alignment is displayed in Figure S1.

### Sequence Analysis

All alignments were produced using ClustalX 2.0.9 (ftp://ftp.ebi.ac.uk/pub/software/clustalw2/). Blast 2.0.14 was obtained from NCBI and the program “blastall” was used with default options, and the Blosum 62 scoring matrix. *Nostoc sp.* (NCBI Geninfo identifier 24637372) was used as a query sequence for “blastall” to search against each of the other NifD sequences in Table 1. In addition to “E-values”, information content is reported (in bits) for this analysis and displayed in Figure 1. Covariant residues in subfamilies were identified in the complete list of 69 nitrogenases by the strong motif algorithm described in [4].

### Phylogenetic Analysis

All phylogenetic analysis were performed using Phylip 3.63 (http://evolution.genetics.washington.edu/phylip.html). The phylogeny in Figure 2 is based on a consensus tree from 100 bootstrap samples using the “protdist” function and neighboring-joining algorithm in Phylip using the default options. The phylogeny image was created using the Phylip “drawgram” function available at http://mobyle.pasteur.fr/. The scale bar and box outline were inserted into the image using the Microsoft Paint software, which was also used to re-draw several branch labels that overlapped. Figure 3 was based on editing Figure 2 in Microsoft PowerPoint to highlight sub-classes of nitrogenases in our analysis.

## Supporting Information

Figure S1 Alignment of nitrogenases. Sequences are based on identifiers which include a numbering scheme followed by the species name. A legend associating these identifiers with the organism name and GI number are in Supporting Table S4. Coloring of similar residues are based on the default ClustalX color parameter file. At the bottom of the alignment, a plot shows the level of conservation at each position indicated by the height of the bar. At the top of the alignment, the symbol * indicates a fully conserved position, “:” indicates that a “strong” residue groups is conserved, while “. ” indicates that a “weak” residue group is conserved (see ClustalX manual for details).
(1.37 MB PDF)

Table S1 Lineages of Bacteria with Group I NifD sequences listed in Table 1.
(0.04 MB DOC)

Table S2 Lineages of Archaea and Bacteria with Group 2 NifD sequences listed in Table 2.
(0.03 MB DOC)

Table S3 Regions of the NifD sequences in different *Clostridium* strains flanking the His and Cys residues that attach the FeMo cofactor to the protein.
(0.04 MB DOC)

Table S4 Legend for multiple alignment and phylogenies.
(0.11 MB DOC)
