# Supplementary material for: Conserved Amino Acid Sequence Features in the α Subunits of MoFe, VFe, and FeFe Nitrogenases
Source: PLoS One. 2009 Jul 3;4(7):e6136. doi: 10.1371/journal.pone.0006136 (PMC2700964; doi:10.1371/journal.pone.0006136)
Supplement: Figure S1 — Alignment of nitrogenases. Sequences are based on identifiers which include a numbering scheme followed by the species name. A legend associating these identifiers with the organism name and GI number are in Supporting Table S4. Coloring of similar residues are based on the default ClustalX color parameter file. At the bottom of the alignment, a plot shows the level of conservation at each position indicated by the height of the bar. At the top of the alignment, the symbol * indicates a fully conserved position, “:” indicates that a “strong” residue groups is conserved, while “. ” indicates that a “weak” residue group is conserved (see ClustalX manual for details). (1.37 MB PDF) [file pone.0006136.s001.pdf]

Figure S1

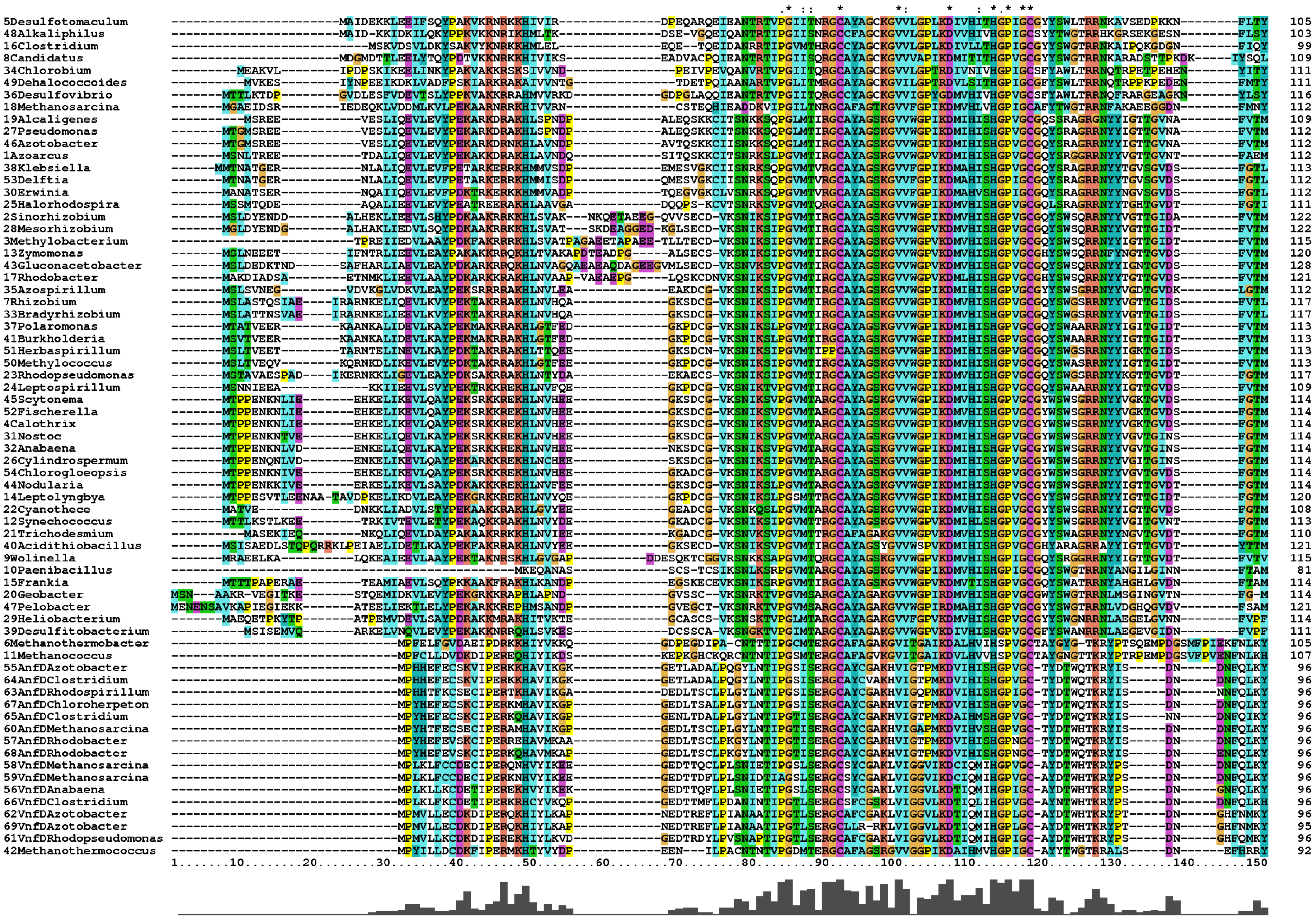

5Desulfotomaculum
48Alkaliphilus
16Clostridium
8Candidatus
34Chlorobium
49Dehalococcoides
36Desulfovibrio
18Methanosarcina
19Alcaligenes
27Pseudomonas
46Azotobacter
1Azoarcus
38Klebsiella
53Delftia
30Erwinia
25Halorhodospira
2Sinorhizobium
28Mesorhizobium
3Methylobacterium
13Zymomonas
43Gluconacetobacter
17Rhodobacter
35Azospirillum
7Rhizobium
33Bradyrhizobium
37Polaromonas
41Burkholderia
51Herbaspirillum
50Methylococcus
23Rhodopseudomonas
24Leptospirillum
45Scytonema
52Fischerella
4Calothrix
31Nostoc
32Anabaena
26Cylindrospermum
54Chlorogloeopsis
44Nodularia
14Leptolyngbya
22Cyanothece
12Synechococcus
21Trichodesmium
40Acidithiobacillus
9Wolinella
10Paenibacillus
15Frankia
20Geobacter
47Pelobacter
29Heliobacterium
39Desulfitobacterium
6Methanothermobacter
11Methanococcus
55AnfDAzotobacter
64AnfDClostridium
63AnfDRhodospirillum
67AnfDChloroherpeton
65AnfDClostridium
60AnfDMethanosarcina
57AnfDRhodobacter
68AnfDRhodobacter
58VnfDMethanosarcina
59VnfDMethanosarcina
56VnfDAnabaena
66VnfDClostridium
62VnfDAzotobacter
69VnfDAzotobacter
61VnfDRhodopseudomonas
42Methanothermococcus

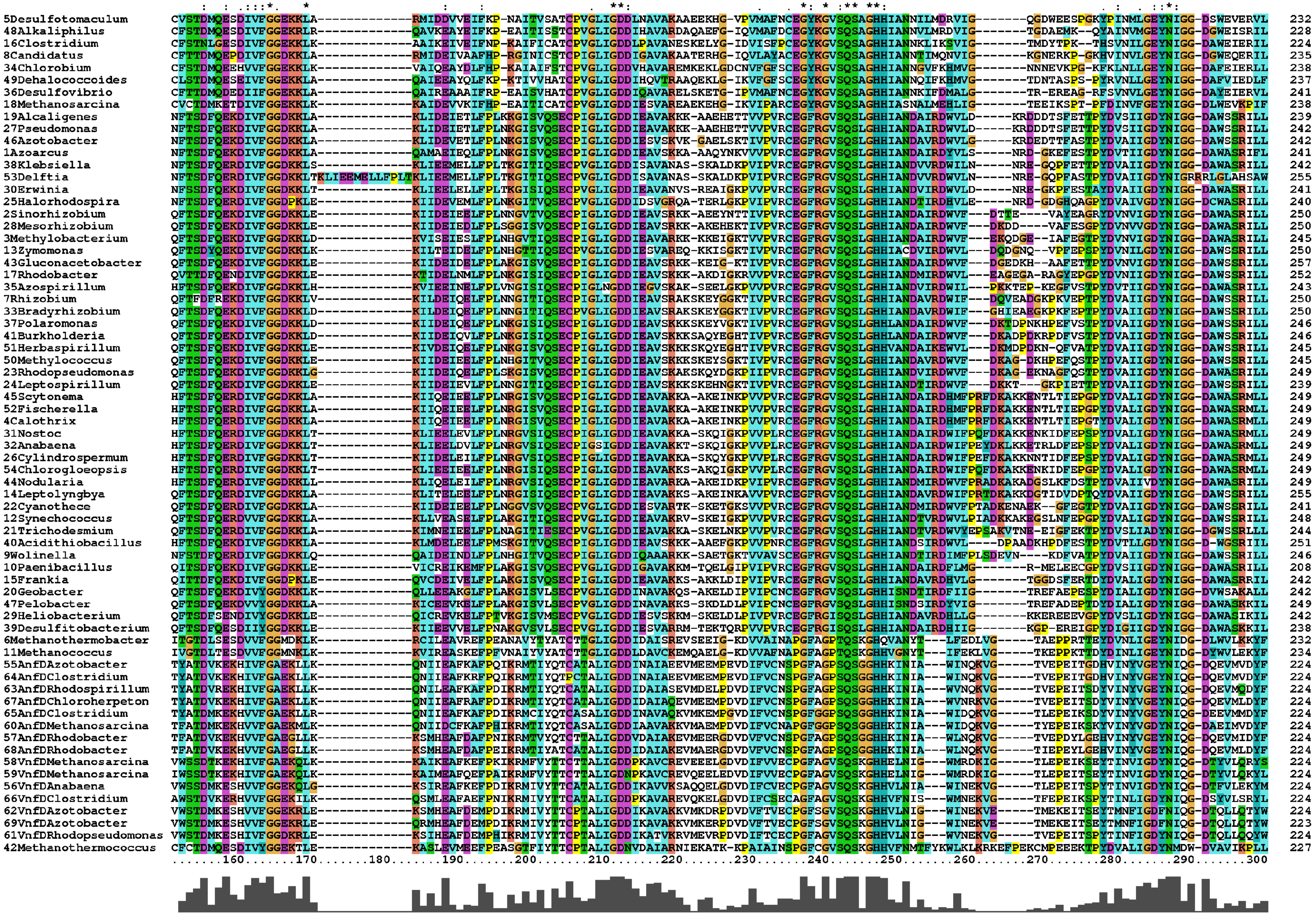
5Desulfotomaculum 232
48Alkaliphilus 228
16Clostridium 224
8Candidatus 235
34Chlorobium 238
49Dehalococcoides 237
36Desulfovibrio 241
18Methanosarcina 238
19Alcaligenes 239
27Pseudomonas 242
46Azotobacter 242
1Azoarcus 241
38Klebsiella 242
53Delftia 255
30Erwinia 241
25Halorhodospira 240
2Sinorhizobium 250
28Mesorhizobium 250
3Methylobacterium 245
13Zymomonas 250
43Gluconacetobacter 257
17Rhodobacter 252
35Azospirillum 243
7Rhizobium 250
33Bradyrhizobium 250
37Polaromonas 246
41Burkholderia 246
51Herbaspirillum 245
50Methylococcus 245
23Rhodopseudomonas 249
24Leptospirillum 239
45Scytonema 249
52Fischerella 249
4Calothrix 249
31Nostoc 249
32Anabaena 249
26Cylindrospermum 249
54Chlorogloeopsis 249
44Nodularia 249
14Leptolyngbya 255
22Cyanothece 241
12Synechococcus 248
21Trichodesmium 244
40Acidithiobacillus 251
9Wolinella 246
10Paenibacillus 208
15Frankia 242
20Geobacter 242
47Pelobacter 249
29Heliobacterium 242
39Desulfitobacterium 238
6Methanothermobacter 232
11Methanococcus 234
55AnfDAzotobacter 224
64AnfDClostridium 224
63AnfDRhodospirillum 224
67AnfDChloroherpeton 224
65AnfDClostridium 224
60AnfDMethanosarcina 224
57AnfDRhodobacter 224
68AnfDRhodobacter 224
58VnfDMethanosarcina 224
59VnfDMethanosarcina 224
56VnfDAnabaena 224
66VnfDClostridium 224
62VnfDAzotobacter 224
69VnfDAzotobacter 223
61VnfDRhodopseudomonas 224
42Methanothermococcus 227
160 170 180 190 200 210 220 230 240 250 260 270 280 290 300

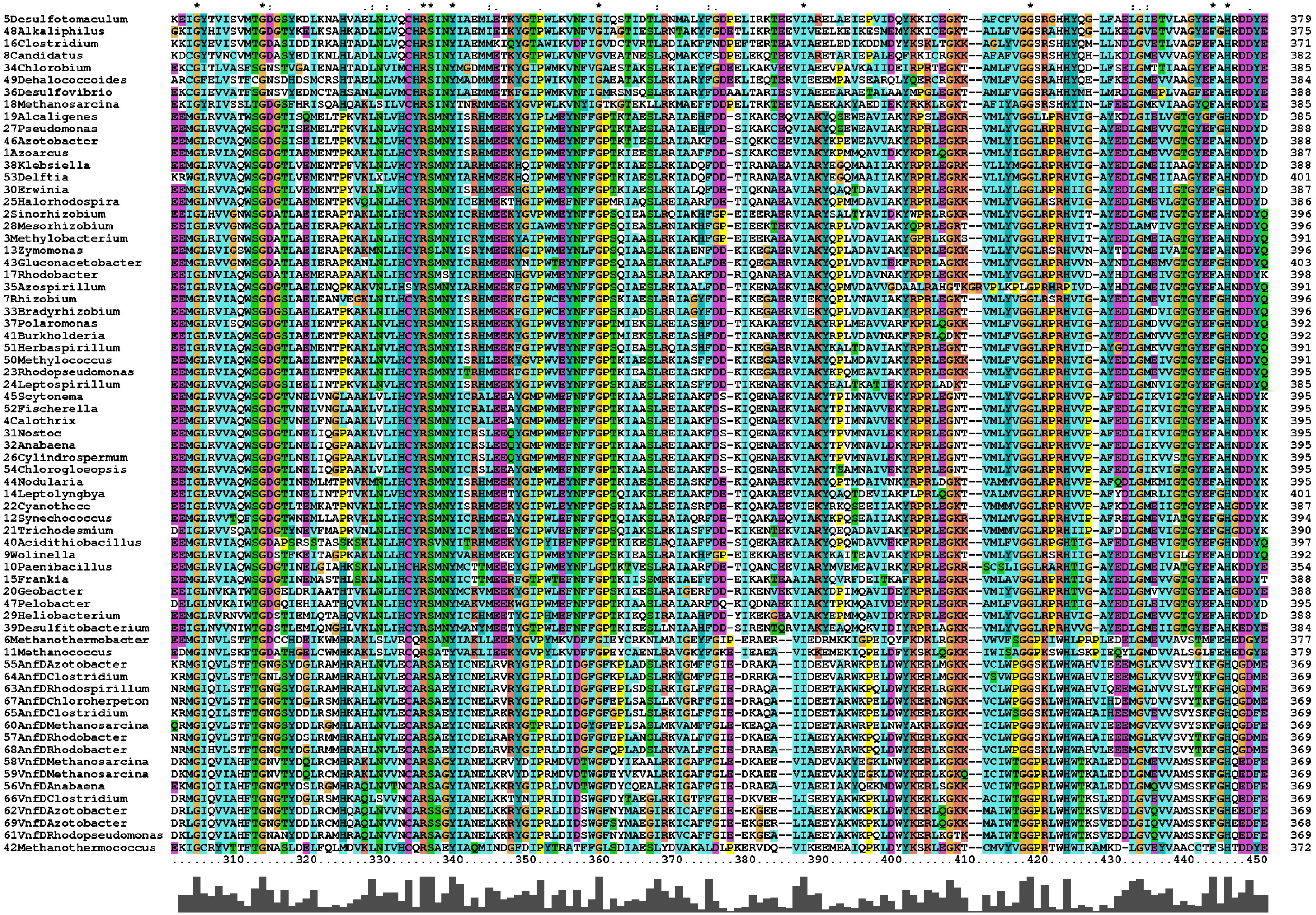

5Desulfotomaculum 379
48Alkaliphilus 375
16Clostridium 371
8Candidatus 382
34Chlorobium 385
49Dehalococcoides 384
36Desulfovibrio 388
18Methanosarcina 385
19Alcaligenes 385
27Pseudomonas 388
46Azotobacter 388
1Azoarcus 387
38Klebsiella 388
53Delftia 401
30Erwinia 387
25Halorhodospira 386
2Sinorhizobium 396
28Mesorhizobium 396
3Methylobacterium 391
13Zymomonas 396
43Gluconacetobacter 403
17Rhodobacter 398
35Azospirillum 391
7Rhizobium 396
33Bradyrhizobium 396
37Polaromonas 392
41Burkholderia 392
51Herbaspirillum 391
50Methylococcus 391
23Rhodopseudomonas 395
24Leptospirillum 385
45Scytonema 395
52Fischerella 395
4Calothrix 395
31Nostoc 395
32Anabaena 395
26Cylindrospermum 395
54Chlorogloeopsis 395
44Nodularia 395
14Leptolyngbya 401
22Cyanothece 387
12Synechococcus 394
21Trichodesmium 390
40Acidithiobacillus 397
9Wolinella 392
10Paenibacillus 354
15Frankia 388
20Geobacter 388
47Pelobacter 395
29Heliobacterium 388
39Desulfitobacterium 384
6Methanothermobacter 377
11Methanococcus 379
55AnfDAzotobacter 369
64AnfDClostridium 369
63AnfDRhodospirillum 369
67AnfDChloroherpeton 369
65AnfDClostridium 369
60AnfDMethanosarcina 369
57AnfDRhodobacter 369
68AnfDRhodobacter 369
58VnfDMethanosarcina 369
59VnfDMethanosarcina 369
56VnfDAnabaena 369
66VnfDClostridium 369
62VnfDAzotobacter 369
69VnfDAzotobacter 368
61VnfDRhodopseudomonas 369
42Methanothermococcus 372
310 320 330 340 350 360 370 380 390 400 410 420 430 440 450

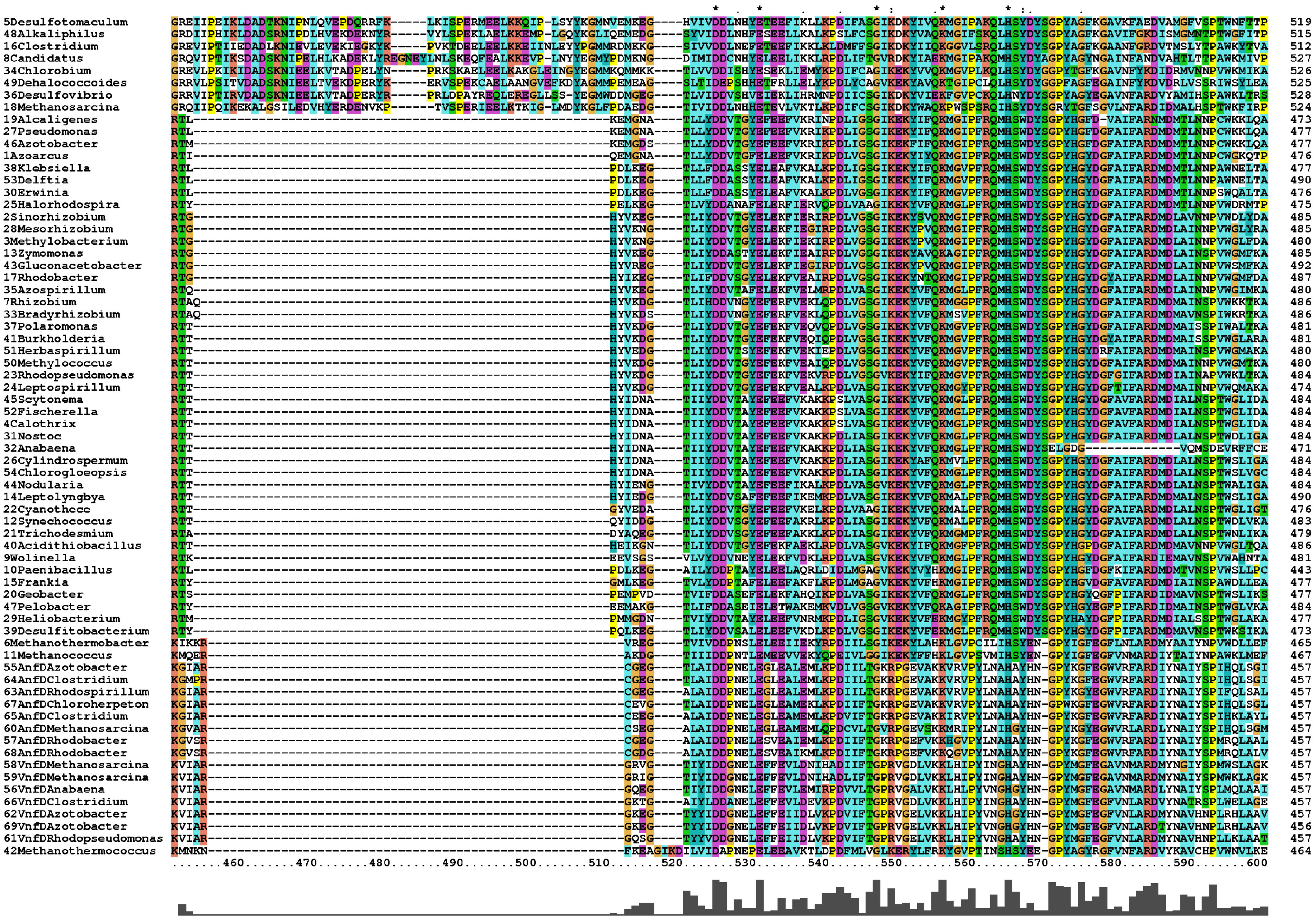

```
5Desulfotomaculum        PWKN--KPIIEGTIEEGGCSKC------------------------------------------------------------------------------------------------------------ 539
48Alkaliphilus           PWKQ--DALLEGEVIEE-VATC------------------------------------------------------------------------------------------------------------ 534
16Clostridium            PWKT--ESILEGTFGGEV---------------------------------------------------------------------------------------------------------------- 528
8Candidatus              PWER--IEEPDTGKQEGANDA------------------------------------------------------------------------------------------------------------- 546
34Chlorobium             PWEKSEPESLEASYVAS----------------------------------------------------------------------------------------------------------------- 543
49Dehalococcoides        PWQR--QTQVKATYGI------------------------------------------------------------------------------------------------------------------ 539
36Desulfovibrio          PFDT--GPTLTAGLND------------------------------------------------------------------------------------------------------------------ 542
18Methanosarcina         PWKA---ENVE----------------------------------------------------------------------------------------------------------------------- 532
19Alcaligenes            PWEKAEESAEKVAASA------------------------------------------------------------------------------------------------------------------ 489
27Pseudomonas            PWQKAEESAEKVAASA------------------------------------------------------------------------------------------------------------------ 493
46Azotobacter            PWEASEG-AEKVAASA------------------------------------------------------------------------------------------------------------------ 492
1Azoarcus                PWKKTEATEDVKAAA------------------------------------------------------------------------------------------------------------------- 491
38Klebsiella             PWLKSA---------------------------------------------------------------------------------------------------------------------------- 483
53Delftia                PWLKSA---------------------------------------------------------------------------------------------------------------------------- 496
30Erwinia                PWLKSA---------------------------------------------------------------------------------------------------------------------------- 482
25Halorhodospira         PWKATGEPASKAA--------------------------------------------------------------------------------------------------------------------- 488
2Sinorhizobium           PWQKAAMLAASGAAE------------------------------------------------------------------------------------------------------------------- 500
28Mesorhizobium          PWKKMKDAPLRAVAAE------------------------------------------------------------------------------------------------------------------ 501
3Methylobacterium        PWKAKPAPAFLDAAE------------------------------------------------------------------------------------------------------------------- 495
13Zymomonas              PWKTAA---------------------------------------------------------------------------------------------------------------------------- 491
43Gluconacetobacter      PWKNAA---------------------------------------------------------------------------------------------------------------------------- 498
17Rhodobacter            PWKKTA---------------------------------------------------------------------------------------------------------------------------- 493
35Azospirillum           PF-------------------------------------------------------------------------------------------------------------------------------- 482
7Rhizobium               PWKEASRAKLLAAEMAQSAEHVLD---------------------------------------------------------------------------------------------------------- 510
33Bradyrhizobium         PWKEAPSAKLQAAE-------------------------------------------------------------------------------------------------------------------- 500
37Polaromonas            PWKKAA---------------------------------------------------------------------------------------------------------------------------- 487
41Burkholderia           PWKKTA---------------------------------------------------------------------------------------------------------------------------- 487
51Herbaspirillum         PWKA------------------------------------------------------------------------------------------------------------------------------ 484
50Methylococcus          PWKSAA---------------------------------------------------------------------------------------------------------------------------- 486
23Rhodopseudomonas       PWS------------------------------------------------------------------------------------------------------------------------------- 487
24Leptospirillum         PF-------------------------------------------------------------------------------------------------------------------------------- 476
45Scytonema              PWNKKEAKAKVA---------------------------------------------------------------------------------------------------------------------- 496
52Fischerella            PWNKKEAKAKVA---------------------------------------------------------------------------------------------------------------------- 496
4Calothrix               PWNKKEAKAKVA---------------------------------------------------------------------------------------------------------------------- 496
31Nostoc                 PWKKAATKAKAAA--------------------------------------------------------------------------------------------------------------------- 497
32Anabaena               GRKKSLFLA------------------------------------------------------------------------------------------------------------------------- 480
26Cylindrospermum        PWKKAATKAKAAA--------------------------------------------------------------------------------------------------------------------- 497
54Chlorogloeopsis        SLEKVRS--------------------------------------------------------------------------------------------------------------------------- 491
44Nodularia              PWNKKAQAKKAKAKQQPREIAKG----------------------------------------------------------------------------------------------------------- 507
14Leptolyngbya           PWSK------------------------------------------------------------------------------------------------------------------------------ 494
22Cyanothece             PWNK------------------------------------------------------------------------------------------------------------------------------ 480
12Synechococcus          PWKS------------------------------------------------------------------------------------------------------------------------------ 487
21Trichodesmium          PWKQAK---------------------------------------------------------------------------------------------------------------------------- 485
40Acidithiobacillus      PWK------------------------------------------------------------------------------------------------------------------------------- 489
9Wolinella               PWD------------------------------------------------------------------------------------------------------------------------------- 484
10Paenibacillus          REKVEVSV-------------------------------------------------------------------------------------------------------------------------- 451
15Frankia                PWSKAGEVA------------------------------------------------------------------------------------------------------------------------- 486
20Geobacter              PF-------------------------------------------------------------------------------------------------------------------------------- 479
47Pelobacter             PF-------------------------------------------------------------------------------------------------------------------------------- 486
29Heliobacterium         PWKKETVKEVR----------------------------------------------------------------------------------------------------------------------- 488
39Desulfitobacterium     PWMK------------------------------------------------------------------------------------------------------------------------------ 477
6Methanothermobacter     EEDVN----------------------------------------------------------------------------------------------------------------------------- 470
11Methanococcus          EEEEPGDSNE------------------------------------------------------------------------------------------------------------------------ 477
55AnfDAzotobacter        DITKDNAPEWGNGFRTRQMLSDGNLSDAVRNSETLRQYTGGYDSVSKLREREYPAFERKVG--------------------------------------------------------------------- 518
64AnfDClostridium        DITKDNAPEWGNGFRTRQMLSDGNLSDAVRNSETLAQYTGGYDSVSNVREREYPAFERKVG--------------------------------------------------------------------- 518
63AnfDRhodospirillum     DISKDPIPT-DQGFLTPQMISDPALPAEVRSSTVLTPYRGAYDTISALREKTYPRFDAVPVAQ------------------------------------------------------------------- 519
67AnfDChloroherpeton     DISKDEIPT-EHGFVTQRMISDATLSHEVRTSAMVREYTGKYDPIADLRKKTYPDLKK------------------------------------------------------------------------ 514
65AnfDClostridium        DISKDEIPT-DKGFSTQKMISDVNLSEEVVSSPDLREYTGKCNIIPDLCKKTYPDFPLKK---------------------------------------------------------------------- 516
60AnfDMethanosarcina     DISKDEIPT-DKGFLTRQMISDVNIVDDRTTPSEERPYTGDWDIVTRLRGKTYPKLESQQLGTA------------------------------------------------------------------ 520
57AnfDRhodobacter        DISAPDAAI-TSGFRTAKMNADLTVSDEVKFSEVLHEYTGKYDSIAEIRARNQPMPPSRK-LRDAVQPAAE----------------------------------------------------------- 526
68AnfDRhodobacter        DISQPDAAI-TSGFRTAKMNADATLPEEVKFDTRLQQYTGKYDSIAEIRARNLAYAAEQKALRDAERAAAE----------------------------------------------------------- 527
58VnfDMethanosarcina     DPR------------------------------------------------VVQEL-------------------------------------------------------------------------- 465
59VnfDMethanosarcina     DPRETD---------------------------------SPMWSLTEKDSGVVQES-------------------------------------------------------------------------- 480
56VnfDAnabaena           DVRDDAPKAPAKTKEIEHLNEKVTNITTYIQERCLWQFHSRAWDREENINGVIKKAAELLSGERSVQETLTDKLHYADAKILVSELKRNLPWIKELDKAQVKSVLESVKQNLVGIAIAGSLNGELHHSLY 587
66VnfDClostridium        DIREV----------------------------------------------------------------------------------------------------------------------------- 462
62VnfDAzotobacter        DIRDKS----------------------------------------QTTPVIVRGAA------------------------------------------------------------------------- 474
69VnfDAzotobacter        DIRDSS----------------------------------------QTTPVIVRGAA------------------------------------------------------------------------- 473
61VnfDRhodopseudomonas   DIRG------------------------------------------ETSTRLLEAAE------------------------------------------------------------------------- 472
42Methanothermococcus    GEDKFKN-----------------------------------------FKGDLNE--------------------------------------------------------------------------- 478
                         .......610.......620.......630.......640.......650.......660.......670.......680.......690.......700.......710.......720.......730
```
