# Supplementary material for: Conserved Amino Acid Sequence Features in the α Subunits of MoFe, VFe, and FeFe Nitrogenases
Source: PLoS One. 2009 Jul 3;4(7):e6136. doi: 10.1371/journal.pone.0006136 (PMC2700964; doi:10.1371/journal.pone.0006136)
Supplement: Table S1 — Lineages of Bacteria with Group I NifD sequences listed in Table 1. (0.04 MB DOC) [file pone.0006136.s002.doc]

**Table S1. Lineages of Bacteria with Group I NifD sequences listed in Table 1.**

**Organism Class* Family**

_______________________________________________________________________________________

*Azotobacter vinelandii* γ-Proteobacteria Pseudomonadaceae

*Frankia* sp. Actinobacteria Frankineae

*Azoarcus* sp. β-Proteobacteria Rhodocyclaceae *Alcaligenes faecalis* β-Proteobacteria Alcaligenaceae

*Pseudomonas stutzeri* γ-Proteobacteria Pseudomonadaceae

*Klebsiella* *pneumoniae* γ-Proteobacteria Enterobacteriaceae

*Delftia tsuruhatensis* β-Proteobacteria Comamonadaceae

*Erwinia carotovora* γ-Proteobacteria Enterobacteriaceae

*Halorhodospira halophila* γ-Proteobacteria Ectothiorhodospiraceae

*Rhizobium* sp. α-Proteobacteria Rhizobiaceae

*Bradyrhizobium japonicum* α-Proteobacteria Bradyrhizobiaceae

*Methylococcus capsulatus* γ-Proteobacteria Methylococcaceae

*Polaromonas napthalenivorans* β-Proteobacteria Comamonadaceae

*Burkholderia xenovorans*  β-Proteobacteria Bulkolderiaceae

*Leptospirillum ferrooxidans* Nitrospira Nitrospiraceae

*Herbaspirillum seropedicae*  β-Proteobacteria Oxalobacteraceae

*Rhodopseudomonas palustris* α-Proteobacteria Bradyrhizobiaceae

*Sinorhizobium medicae* α-Proteobacteria Rhizobiaceae

*Mesorhizobium loti* α-Proteobacteria Phyllobacteriaceae

*Methylobacterium* sp. α-Proteobacteria Methylobacteriaceae

*Gluconacetobacter diazotrophicus* α-Proteobacteria Acetobacteraceae

*Zymomonas mobilis* α-Proteobacteria Sphingomonadaceae

*Gluconacetobacter diazotrophicus* α-Proteobacteria Acetobcteraceae

*Rhodobacter sphaeroides* α-Proteobacteria Rhodobacteraceae

*Azospirillum brasilense*  α-Proteobacteria Rhodospirillaceae

*Scytonema* sp. Cyanobacteria Scytonemataceae

*Fischerella muscicola* Cyanobacteria

*Calothrix desertica* Cyanobacteria Rivulariaceae

Chlorogloeopsis fritschii Cyanobacteria

*Cylindrospermum majus* Cyanobacteria Nostocaceae

*Nostoc* sp. Cyanobacteria Nostocaceae

*Anabaena* sp. Cyanobacteria Nostocaceae

*Nodularia spumigena* Cyanobacteria Nostocaceae

*Leptolyngbya boryana*  Cyanobacteria

*Synechococcus* sp. Cyanobacteria

*Trichodesmium* *erythraeum* Cyanobacteria

*Cyanothece* sp. Cyanobacteria

*Wolinella succinogenenes* ε-Proteobacteria Helicobacteraceae

*Acidithiobacillus ferrooxidans* γ-Proteobacteria Acidithiobacillaceae

*Paenibacillus massiliensis* Bacilli Paenibacillaceae

*Heliobacterium chlorum* Clostridia Heliobacteraceae

*Desulfitobacterium hafniense* Clostridia Peptococcaceae

*Geobacter sulfurreducens* δ-Proteobacteria Geobacteraceae

*Pelobacter carbinolicus* δ-Proteobacteria Pelobacteraceae

_______________________________________________________________________________________

* Lineage data were obtained from the NCBI Taxonomy Browser at http://www.ncbi.nlm.nih.gov/Taxonomy/Browser/wwwtax.cgi
