# Supplementary material for: Conserved Amino Acid Sequence Features in the α Subunits of MoFe, VFe, and FeFe Nitrogenases
Source: PLoS One. 2009 Jul 3;4(7):e6136. doi: 10.1371/journal.pone.0006136 (PMC2700964; doi:10.1371/journal.pone.0006136)
Supplement: Table S2 — Lineages of Archaea and Bacteria with Group 2 NifD sequences listed in Table 2. (0.03 MB DOC) [file pone.0006136.s003.doc]

**Table S2. Lineages of Archaea and Bacteria with Group 2 NifD sequences listed in Table 2.**

**Class* Family**

_______________________________________________________________________________

**Archaea**

*Methanothermobacter thermautotrophicus* Methanobacteria Methanobacteriaceae

*Methanococcus maripaludis* Methanococci Methanococcaceae

*Methanothermococcus thermolithotrophicus* Methanococci Methanococcaceae

*Candidatus Methanoregula boonei* Methanomicrobia Uncertain

*Methanosarcina barkeri* Methanosarcina Methanosarcinaceae

**Bacteria**

*Desulfotomaculum reducens* Clostridia Peptococcaceae

*Alkaliphilus metalliredigens* Clostridia Clostridiaceae

*Clostridium beijerinckii*  Clostridia Clostridiaceae

*Candidatus Methanoregula boonei* Methanomicrobia Uncertain

*Chlorobium tepidum*  Chlorobia Clorobiaceae

*Dehalococcoides ethenogenes* Dehalococcoidetes Dehalococcoides

*Desulfovibrio vulgaris* δ-Proteobacteria Desulfovibrionaceae

_______________________________________________________________________________

* Lineage data were obtained from the NCBI Taxonomy Browser at http://www.ncbi.nlm.nih.gov/Taxonomy/Browser/wwwtax.cgi
