# Supplementary material for: Conserved Amino Acid Sequence Features in the α Subunits of MoFe, VFe, and FeFe Nitrogenases
Source: PLoS One. 2009 Jul 3;4(7):e6136. doi: 10.1371/journal.pone.0006136 (PMC2700964; doi:10.1371/journal.pone.0006136)
Supplement: Table S3 — Regions of the NifD sequences in different Clostridium strains flanking the His and Cys residues that attach the FeMo cofactor to the protein. (0.04 MB DOC) [file pone.0006136.s004.doc]

**Table S3**. **Regions of the NifD sequences in different *Clostridium* strains flanking the His and Cys residues that attach the FeMo cofactor to the protein.**

**Organism**† **GI* Sequences flanking Cys-275****

*C. kluyveri* DSM 555 153955692 **L**NLVQ**C**H**RS**IN**Y**

*C. acetobutylicum* ATCC 824 15893548 **L**NLVQ**C**H**RS**IN**Y**

*C. butyricum* 5521 182417522 **L**NVIQ**C**H**RS**IN**Y**

*C. beijerinckii* NCIMB 8052 150016874 **L**NLVQ**C**H**RS**IN**Y**

*C. beijerinckii* NRRL B593 46395070 **L**NLVQ**C**H**RS**IN**Y**

*C. pasteurianum* W5 128242/47827220 **L**NLVQ**C**H**RS**IN**Y**

**Sequences flanking His-442****

*C. kluyveri* DSM 555 153955692 SKQL**HSY**DY

*C. acetobutylicum* ATCC 824 15893548 SKQL**HSY**DY

*C. butyricum* 5521 182417522 SRGL**HSY**DY

*C. beijerinckii* NCIMB 8052 150016874 SRQL**HSY**DY

*C. beijerinckii* NRRL B593 46395070 SRQL**HSY**DY

*C. pasteurianum* W5 128242/47827220 SKQL**HSY**DY

^*^ GI is the NCBI Geninfo identifier.

^**^  In these *Clostridium* sequences, residues conserved in all Group II NifD sequences encompassing His **α**-442 or Cys **α**-275 (using *Azotobacter vinelandii*

sequence numbering), shown in Table 2, are in bold face.

† All *Clostridium* sequences examined contained the insertion.
