# Supplementary material for: Conserved Amino Acid Sequence Features in the α Subunits of MoFe, VFe, and FeFe Nitrogenases
Source: PLoS One. 2009 Jul 3;4(7):e6136. doi: 10.1371/journal.pone.0006136 (PMC2700964; doi:10.1371/journal.pone.0006136)
Supplement: Table S4 — Legend for multiple alignment and phylogenies. (0.11 MB DOC) [file pone.0006136.s005.doc]

**Table S4: Legend for multiple alignment and phylogenies.**

| **Protein** | **Identifier** | **GI*** | **Organism** |
| --- | --- | --- | --- |
| NifD | 1Azoarcus | 119669243 | *Azoarcus sp.* |
| NifD | 2Sinorhizobium | 150378166 | *Sinorhizobium medicae* |
| NifD | 3Methylobacterium | 149123939 | *Methylobacterium sp.* |
| NifD | 4Calothrix | 24637368 | *Calothrix desertica* |
| NifD | 5Desulfotomaculum | 134300651 | *Desulfotomaculum reducens* |
| NifD | 6Methanothermobacter | 1854556 | *Methanothermobacter thermautotrophicus* |
| NifD | 7Rhizobium | 224328 | *Rhizobium sp.* |
| NifD | 8Candidatus | 154150688 | *Candidatus Methanoregula* |
| NifD | 9Wolinella | 34483460 | *Wolinella succinogenes* |
| NifD | 10Paenibacillus | 62512189 | *Paenibacillus massiliensis* |
| NifD | 11Methanococcus | 46397844 | *Methanococcus maripaludis* |
| NifD | 12Synechococcus | 86607919 | *Synechococcus sp.* |
| NifD | 13Zymomonas | 56552720 | *Zymomonas mobilis* |
| NifD | 14Leptolyngbya | 228688 | *Leptolyngbya boryana* |
| NifD | 15Frankia | 21930294 | *Frankia sp.* |
| NifD | 16Clostridium | 150016874 | *Clostridium beijerinckii* |
| NifD | 17Rhodobacter | 77464109 | *Rhodobacter sphaeroides* |
| NifD | 18Methanosarcina | 508282 | *Methanosarcina barkeri* |
| NifD | 19Alcaligenes | 1183862 | *Alcaligenes faecalis* |
| NifD | 20Geobacter | 39997913 | *Geobacter sulfurreducens* |
| NifD | 21Trichodesmium | 3372146 | *Trichodesmium sp.* |
| NifD | 22Cyanothece | 2197063 | *Cyanothece sp.* |
| NifD | 23Rhodopseudomonas | 39937677 | *Rhodopseudomonas palustris* |
| NifD | 24Leptospirillum | 31747711 | *Leptospirillum ferrooxidans* |
| NifD | 25Halorhodospira | 62122622 | *Halorhodospira halophila* |
| NifD | 26Cylindrospermum | 30983589 | *Cylindrospermum majus* |
| NifD | 27Pseudomonas | 146281711 | *Pseudomonas stutzeri* |
| NifD | 28Mesorhizobium | 20804122 | *Mesorhizobium loti* |
| NifD | 29Heliobacterium | 62751062 | *Heliobacterium chlorum* |
| NifD | 30Erwinia | 50121879 | *Erwinia carotovora* |
| NifD | 31Nostoc | 24637372 | *Nostoc sp.* |
| NifD | 32Anabaena | 223741 | *Anabaena sp.* |
| NifD | 33Bradyrhizobium | 12620453 | *Bradyrhizobium japonicum* |
| NifD | 34Chlorobium | 21674355 | *Chlorobium tepidum* |
| NifD | 35Azospirillum | 142417 | *Azospirillum brasilense* |
| NifD | 36Desulfovibrio | 46562234 | *Desulfovibrio vulgaris* |
| NifD | 37Polaromonas | 121605243 | *Polaromonas naphthalenivorans* |
| NifD | 38Klebsiella | 43847 | *Klebsiella pneumoniae* |
| NifD | 39Desulfitobacterium | 109648676 | *Desulfitobacterium hafniense* |
| NifD | 40Acidithiobacillus | 154639 | *Acidithiobacillus ferrooxidans* |
| NifD | 41Burkholderia | 91778640 | *Burkholderia xenovorans* |
| NifD | 42Methanothermococcus | 128245 | *Methanothermococcus thermolithotrophicus* |
| NifD | 43Gluconacetobacter | 4103974 | *Gluconacetobacter diazotrophicus* |
| NifD | 44Nodularia | 24637384 | *Nodularia spumigena* |
| NifD | 45Scytonema | 30983593 | *Scytonema sp.* |
| NifD | 46Azotobacter | 758358 | *Azotobacter vinelandii* |
| NifD | 47Pelobacter | 77919694 | *Pelobacter carbinolicus* |
| NifD | 48Alkaliphilus | 150391258 | *Alkaliphilus metalliredigens* |
| NifD | 49Dehalococcoides | 57234132 | *Dehalococcoides ethenogenes* |
| NifD | 50Methylococcus | 53802573 | *Methylococcus capsulatus* |
| NifD | 51Herbaspirillum | 6093493 | *Herbaspirillum seropedicae* |
| NifD | 52Fischerella | 24637390 | *Fischerella muscicola* |
| NifD | 53Delftia | 45269095 | *Delftia tsuruhatensis* |
| NifD | 54Chlorogloeopsis | 24637386 | *Chlorogloeopsis fritschii* |
| AnfD | 55AnfDAzotobacter | 113854 | *Azotobacter vinelandii* |
| VnfD | 56VnfDAnabaena | 416166 | *Anabaena variabilis* |
| AnfD | 57AnfDRhodobacter | 728856 | *Rhodobacter capsulatus* |
| VnfD | 58VnfDMethanosarcina | 8099626 | *Methanosarcina barkeri* |
| VnfD | 59VnfDMethanosarcina | 19915055 | *Methanosarcina acetivorans* C2A |
| AnfD | 60AnfDMethanosarcina | 20090074 | *Methanosarcina acetivorans* C2A |
| VnfD | 61VnfDRhodopseudomonas | 39648301 | *Rhodopseudomonas palustris* CGA009 |
| VnfD | 62VnfDAzotobacter | 67154938 | *Azotobacter vinelandii* AvOP |
| AnfD | 63AnfDRhodospirillum | 83592730 | *Rhodospirillum rubrum* ATCC 11170 |
| AnfD | 64AnfDClostridium | 84028173 | *Clostridium hungatei* |
| AnfD | 65AnfDClostridium | 146345888 | *Clostridium kluyveri* DSM 555 |
| VnfD | 66VnfDClostridium | 153954372 | *Clostridium kluyveri* DSM 555 |
| AnfD | 67AnfDChloroherpeton | 193215536 | *Chloroherpeton thalassium* ATCC 35110 |
| AnfD | 68AnfDRhodobacter | 221369728 | *Rhodobacter sphaeroides* KD131 |
| VnfD | 69VnfDAzotobacter | 138885 | *Azotobacter chroococcum* Mcd1 |

* GI is the NCBI GenInfo identifier.
